# An Improved Method for the Position Detection of a Quadrant Detector for Free Space Optical Communication

**DOI:** 10.3390/s19010175

**Published:** 2019-01-05

**Authors:** Qing Li, Shaoxiong Xu, Jiawei Yu, Lingjie Yan, Yongmei Huang

**Affiliations:** 1 Institute of Optics and Electronics, Chinese Academy of Sciences, No. 1 Guangdian Road, Chengdu 610209, China; qiou9@163.com (Q.L.); hitxsx@163.com (S.X.); yujiawei11@126.com (J.Y.); jeyelche@163.com (L.Y.); 2 School of Optoelectronic Science and Engineering, University of Electronic Science and Technology of China, No. 4 Section 2 North Jianshe Road, Chengdu 610054, China; 3 Key Laboratory of Optical Engineering, Chinese Academy of Sciences, Chengdu 610209, China; 4 University of Chinese Academy of Sciences, Beijing 100049, China

**Keywords:** free space optical communication, quadrant detector, light spot position detection, cyclic cross-correlation

## Abstract

In free space optical communication, a beacon light loses too much energy after a long-distance transmission and faces strong interference from background light. The beacon light illuminated on a quadrant detector (QD) is so weak that the output signal-to-noise ratio (SNR) of a QD is very low, which leads to a significant decrease in the accuracy of the direct position detection method. To solve this problem, an improved light spot position detecting method is proposed. Since the background light and the dark current noise are white noise, we could consider concentrating the energy of QD output signal at a certain frequency point to enhance the output SNR. Therefore, a cosine signal is used to modulate the intensity of a beacon light at the transmitting end. Then the QD output photocurrents are also cosine signals with the same frequency as the modulating signal. Putting the photocurrent signals into a cross-correlation operation with a reference signal, which is the same as the modulating signal, can enhance the QD output SNR at a certain frequency point. Unfortunately, the result of the classical cross-correlation is attenuated with increasing delay. It is hard to detect the amplitude of the classical cross-correlation result. So, we used cyclic cross-correlation to obtain a stable correlation result to detect its amplitude accurately. The experiment results show that even when the QD output SNR is less than −17 dB, the detection root-mean-square error of the proposed method is 0.0092 mm, which is a quarter of the direct position detection method. Moreover, this method only needs a small amount of data to accomplish the calculation and is especially suitable for real-time spot position detection.

## 1. Introduction

Free space optical (FSO) communication is more widely used because it is compatible with the advantages of wireless communication and optical fiber communication. In this communication mode, a laser beam is used to realize point-to-point transmission. The two ends of the communication need to keep aiming and tracking each other to keep the communication link unimpeded. Therefore, a telescope control system is the key subsystem of FSO. The light spot position detection is used to detect the angular deviation of the beacon light in the telescope control subsystem. The accuracy of the light spot position detection determines the performance of the FSO systems.

The FSO system introduced in this article uses the quadrant detector (QD) to detect the beacon light spot position. A QD is widely used in high-accuracy position measurement and tracking because of its advantages, including high resolution, low natural noise, and fast response speed. It is necessary to study how to improve the detection accuracy of a QD. Zhang et al. [1] proposed that the detection accuracy of a QD was related to the spot radius, spot position, and the QD output signal-to-noise ratio (SNR). Wu et al. [2] analyzed the nonlinear characteristic of a QD and proposed a linear correction method based on Boltzmann function. Gao [3] calibrated the real spot position and the ratio of the photocurrent output from a QD and then established a database. However, establishing a database requires a large amount of data, which requires a lot of hardware resources. Cui S. et al. [4,5] expanded the estimated spot position value at the detection spot position value by using the Taylor expansion principle and gave the expression for the spot solution at different levels of linearity. Chen, Silva and Hermosa et al. [6,7,8] calibrated the estimated value of spot position and the real spot position value and then fitted the calibrated value to a polynomial.

All the above methods directly detect the amplitudes of the QD output photocurrents and improve the QD detection accuracy by correcting the nonlinearity of the QD. However, when the QD output SNR is too low, the above methods are not effective. In addition, when light spot radius changes, the above methods need to be recalibrated. Therefore, we propose a method to improve the spot detection accuracy by cyclic cross-correlation which can enhance the QD output SNR at a frequency point. First, we use a cosine signal to modulate the intensity of a beacon light at the transmitting end. Hence, at the receiving end, the energy of the QD output signal is concentrated at a certain frequency point. According to the working principle of lock-in amplifier [9] and the QD, we simplify the phase shift and phase sensitive detection of the lock-in amplifier and directly carry out the cross-correlation operation to extract useful signals. So, a reference signal with the same frequency as the modulating signal is used to cyclic cross-correlation operation with the QD output signals. Finally, we detect the amplitude of the cyclic cross-correlation result and calculate the spot position. This method can greatly improve the position detection accuracy of the QD in the condition of extremely low SNR. Additionally, the cyclic cross-correlation only needs a small amount of data to accomplish the calculation.

## 2. QD Performance Analysis

### 2.1. System Description and Operating Principle of QD

**Figure 1:**
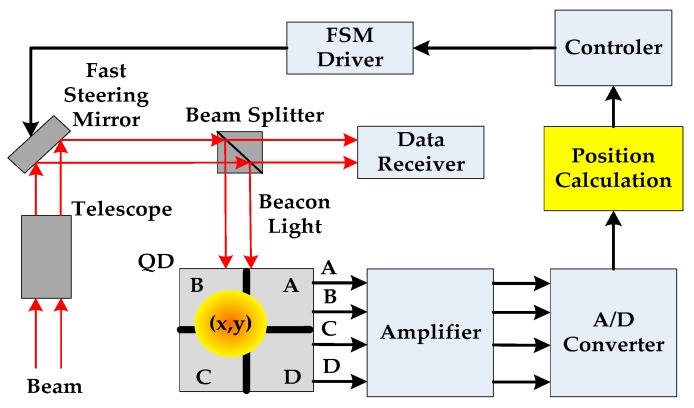
The telescope control system of the free space optical communication (FSO).

FSO communication systems offer greater capabilities than radio frequency systems, but bring greater challenges in implementation. One of the challenges involves the difficulty of acquiring, tracking, and pointing a concentrated beam of laser arriving from another platform after long-distance transmission. One of the methods of tracking between optical communication terminals (OCT) includes the use of a telescope control subsystem with a quadrant detector sensor on each OCT. A block diagram of the telescope control subsystem is shown in Figure 1.

As shown in Figure 1, the QD consists of four identical p-n junction photodiodes. When QD receives the beacon light, each quadrant can independently output photocurrent of which amplitude corresponding to the energy of the beacon light illuminates each quadrant respectively. Therefore, the beacon light position can be estimated according to the ratio of the photocurrents’ amplitudes.

When the center position of the beacon spot is [*x*_0_, *y*_0_], the dimensionless normalized deviation of the Gaussian laser center on the X-axis and Y-axis are, respectively, expressed as the following equation [10,11]:

(1) $$\Delta x=\frac{\left(I_{A}+I_{D})-(I_{B}+I_{C}\right)}{I_{A}+I_{B}+I_{C}+I_{D}};\;\Delta y=\frac{\left(I_{A}+I_{B})-(I_{C}+I_{D}\right)}{I_{A}+I_{B}+I_{C}+I_{D}}$$

where $I_{A}$, $I_{B}$, $I_{C}$, and $I_{D}$ are respectively the photocurrent output from four quadrants in the QD.

### 2.2. QD Detection Error Analysis

In practical application, under the influence of background light and dark current, the photocurrent output from the QD in all quadrants inevitably contains noise components. Therefore, taking the x-axis direction as an example, Equation (1) can be expressed as:

(2) $$\Delta x=\frac{\left(L+L_{n})-(T+T_{n}\right)}{L+L_{n}+T+T_{n}}=(\frac{L-T}{L+T}+\frac{L_{n}-T_{n}}{L+T})\cdot \frac{1}{\left(1+\frac{L_{n}+T_{n}}{L+T}\right)}$$

where $L=I_{A}+I_{D}$; $T=I_{B}+I_{C}$; $L_{n}=I_{nA}+I_{nD}$; $T_{n}=I_{nB}+I_{nC}$. $I_{nA}$, $I_{nB}$, $I_{nC}$ and $I_{nD}$ are noise currents of channel A, B, C, and D, respectively. Taylor expansion of $\frac{1}{\left(1+\frac{L_{n}+T_{n}}{L+T}\right)}$ is carried out and the first two terms are taken. Then, Equation (2) can be rewritten as:

(3) $$\Delta x\approx \frac{L-T}{L+T}+2\cdot \frac{L_{n}T-LT_{n}}{{\left(L+T\right)}^{2}}$$

where the second term is a random error, and its variance is:

(4) $$\sigma_{\Delta x}^{2}=\frac{4L^{2}}{{\left(L+T\right)}^{4}}\sigma_{L}^{2}+\frac{4T^{2}}{{\left(L+T\right)}^{4}}\sigma_{T}^{2}$$

Since the light-sensitive diodes and circuits in the four quadrants of the QD are similar, and the energy of the background light in the four quadrants is equally distributed, $\sigma_{L}^{2}=\sigma_{T}^{2}={\bar{I}}_{n}^{2}$. Thus, we can get:

(5) $$\sigma_{\Delta x}^{2}=\frac{8(L^{2}+T^{2})}{{\left(L+T\right)}^{4}}\cdot {\bar{I}}_{n}^{2}$$

$4\cdot {\bar{I}}_{n}^{2}$ is the total noise power and ${\left(L+T\right)}^{2}$ is the total signal power, therefore, $SNR=\frac{{\left(L+T\right)}^{2}}{4\cdot {\bar{I}}_{n}^{2}}$ is the total power SNR. Make $\alpha =\frac{2\cdot (L^{2}+T^{2})}{{\left(L+T\right)}^{2}}$, then Equation (5) can be written as:

(6) $$\sigma_{\Delta x}^{2}=\frac{\alpha }{SNR}$$

The deviation of Gaussian laser center on the X-axis can also be written as Equation (7):

(7) $$\Delta x=erf(\frac{\sqrt{2}x_{0}}{\omega })$$

where *ω* is the light spot radius and *erf*(·) is the error function. With Equation (7), Equation (1), and $\alpha =\frac{2\cdot (L^{2}+T^{2})}{{\left(L+T\right)}^{2}}$, we can obtain:

(8) $$\alpha =1+erf^{2}(\frac{\sqrt{2}x_{0}}{\omega })$$

Then, the variance of ∆*x* can be written as:

(9) $$\sigma_{\Delta x}^{2}=\frac{1+erf^{2}(\frac{\sqrt{2}x_{0}}{\omega })}{SNR}$$

According to Equation (9), in order to improve the position detection accuracy of the QD, the output SNR of the QD must be improved. However, in practical applications, beacon signal will be greatly attenuated after a long-distance channel. When beacon photocurrents were drowned by noise, it was difficult to detect spot position by using the direct detection method. Therefore, we proposed a high-accuracy light position detection method by enhancing the output SNR of the QD.

## 3. Method for Improving Position Detection Accuracy of QD

The main factors that decrease spot position detection accuracy of the QD are background light and dark current noise, both of which are white noise, and the noise power is distributed in the entire frequency band on average [12,13]. Since the beacon light is modulated by a signal with a single known frequency in the transmitter, the main energy of the photocurrent signal output from QD is concentrated at the known frequency point. Therefore, we can use cyclic cross-correlation algorithm to enhance the SNR of the QD output photocurrent signal at a certain frequency point. Then the photocurrent amplitudes can be accurately measured. Substituting the amplitude values in Equation (1), we can calculate the position of the beacon light spot.

### 3.1. Modulation of Beacon Light

After applying intensity modulation to the beacon in the transmitter, beacon light field is $E_{s}$, and the average power of light field is $P_{s}$:

(10) $$E_{s}(t)=A[1+d(t)]\cos wt$$

(11) $$P_{s}=\bar{E_{s}^{2}(t)}=\frac{A^{2}}{2}[1+2d(t)]$$

where *A* is the intensity of the beacon light and d(t) is the modulating signal. The output photocurrent of the QD corresponding to the beacon light is:

(12) $$I_{beam}=\frac{e\eta }{2hv}A^{2}[1+2d(t)]$$

where $c=\frac{e\eta }{hv}$ is the photoelectric conversion coefficient. As can be seen from Equation (12), the photocurrent has the same frequency as d(t). If d(t) is a narrow band signal, the energy of the photocurrent signal is concentrated at a certain frequency point. A typical narrowband signal is a cosine signal, so d(t) can be defined as $d(t)=\cos(w_{1}t+\varphi_{1})$.

### 3.2. Correlation Detection for Weak Photocurrent Signal

Through the analysis in Section 3.1, the photocurrent $I_{beam}$ output from the QD can be rewritten as a voltage signal:

(13) $$x(t)=KA\cos(w_{1}t+\varphi_{1})+n(t)$$

where *K* is the gain of the amplitude between the QD output port and A/D converter input port, A is the signal amplitude, and n(t) is noise. A reference signal $y(t)=\cos(w_{1}t+\varphi_{2})$ with the same frequency $w_{1}$ is generated by the receiver. Therefore, y(t) has a good time correlation with the signal output from QD, but no time correlation with the noise n(t). By using the cross-correlation operation, the amplitude of x(t) can be accumulated in the time domain, but the amplitude of the noise will not be accumulated. In this way, the SNR of x(t) at the frequency point $w_{1}$ can be enhanced. The cross-correlation operation process of y(t) and x(t) can be written as:

(14) $$\begin{array}{ll} R(\tau ) & =E[x(t)\cdot y(t+\tau )]\\ & =E\{KA\cos(w_{1}t+\varphi_{1})\cdot \cos[w_{1}(t+\tau )]\}+E\{n(t)\cdot \cos[w_{1}(t+\tau )]\}\\ & =R_{xy}(\tau )+R_{ny}(\tau ) \end{array}$$

where E[⋅] represents a mathematical expectation, and the first part of Equation (14) is written as:

(15) $$\begin{array}{ll} R_{xy}(\tau ) & =\underset{T\to \infty }{\lim}\frac{1}{T}\int_{0}^{T}KA\cos(w_{1}t+\varphi_{1})\cdot \cos[w_{1}(t+\tau )]d_{t}\\ & =\underset{T\to \infty }{\lim}\frac{KA}{2T}\int_{0}^{T}\cos[w_{1}(2t+\tau )+\varphi_{1}]d_{t}+\frac{KA}{2}\cos(w_{1}\tau ) \end{array}$$

When T→∞, $\underset{T\to \infty }{\lim}\frac{KA}{2T}\int_{0}^{T}\cos[w_{1}(2t+\tau )+\varphi_{1}]d_{t}=0$, so we can get:

(16) $$R_{xy}(\tau )=\frac{KA}{2}\cos(w_{1}\tau )$$

The second part of Equation (14) is written as:

(17) $$R_{ny}(\tau )=E\{n(t)\cdot \cos[w_{1}(t+\tau )]\}$$

y(t) and n(t) are independent of each other, so the value of $R_{ny}$ is very small. Therefore, $R_{ny}$ can be denoted as a new noise $n_{2}(t)$. Then the final result of Equation (14) can be written as:

(18) $$R(\tau )=r(t)=\frac{KA}{2}\cos(w_{1}t)+n_{2}(t)$$

It can be seen from Equation (18) that the cross-correlation operation can effectively suppress the noise, and the amplitude value *A* can be accurately detected. Substituting *A* into Equation (1) can obtain the position of the light spot on the QD.

### 3.3. Cyclic Cross-Correlation

In Equation (15), only when the time of the correlation operation is infinitely long (T→∞), we can obtain Equations (16) and (18). This means that to accurately estimate the amplitude of R(τ), we need an infinite amount of sampling data to use classical cross-correlation. However, in practical applications, sampling data is truncated. Then, the classical cross-correlation operation after sampling is expressed as:

(19) $$\hat{r}(m)=\frac{1}{N}\sum_{n=0}^{N-1-\left|m\right|}x(n+m)y(n)$$

**Figure 2:**
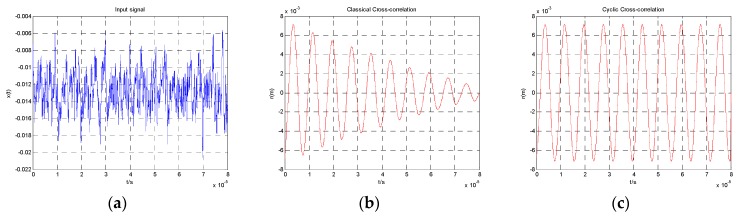
Results of the two kinds of correlation operations. (**a**) Input signal; (**b**) result of classical correlation operation; (**c**) result of the cyclic cross-correlation operation.

The sampling point after truncation is *N*, so the data involved in the operation is only N−|m| points. For each delay *m*, after the multiplication and addition operation, the right-side data of x(n) is removed, and 0 is added into the left side of x(n). With the increase of *m*, the valid data of x(n) decreases. Therefore, as shown in Figure 2b, the $\hat{r}(m)$ amplitude decreases. It is hard to detect the $\hat{r}(m)$ amplitude accurately.

**Figure 3:**
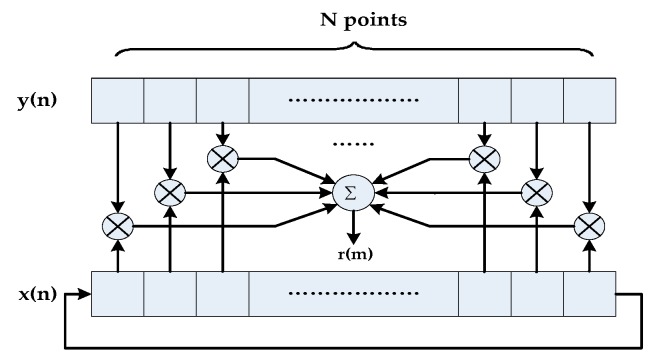
Cyclic cross-correlation operation.

If a set of infinitely long data was put into cross-correlation, we could obtain the same result of Equation (15). A set of infinitely long sampling data can be manufactured by cyclic shifting right method. As shown in Figure 3, we put the data bits which are removed from the right side of x(n) into the left side. For each delay *m*, the amount of valid data involved in cross-correlation is always *N* [14]. When the sampling time is an integer multiple of the period of x(n), the result of r(m) is a cosine signal, and its amplitude is stable, as Figure 2c shows. If x(n) and y(n) have the same frequency, the amplitudes of x(n) at the certain frequency are accumulated in multiple periods, and the noise will be suppressed. Therefore, the SNR of x(n) at the frequency point is enhanced.

As can be seen from Figure 2c, the output result r(m) of cyclic cross-correlation is a stable low-noise cosine signal with the frequency of $w_{1}$. We can detect the r(m) amplitude more accurately.

In order to verify the improvement of the QD output SNR, we sampled the QD output signal in different SNR conditions when the light spot radius ω = 0.53 mm. Then the sampling data with different lengths was substituted into cyclic cross-correlation to obtain the SNR of *r*(*m*). Table 1 recorded the changes of SNR after cyclic cross-correlation, in which T represents how many cycles of sampling data have been intercepted.

As shown in Table 1, as the length of the sampling data involved in the operation increases, the SNR improves greatly. However, when the data length is more than 10 cycles, the improvement of SNR is not obvious.

## 4. Digital Processing of QD

**Figure 4:**
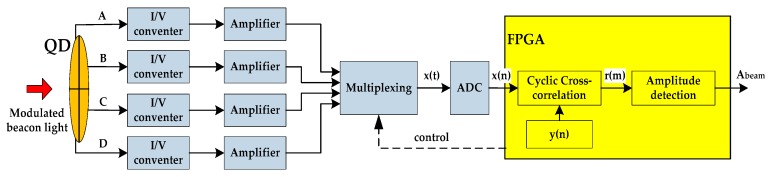
Schematic circuit diagram of digital processing.

The light spot position detection of a QD by using cyclic cross-correlation can be implemented by the system shown in Figure 4 [15,16]. A QD outputs four currents independently, which are converted into voltage signals. After amplification, they are sent to A/D converter (ADC) and converted into digital signals. Then the cyclic cross-correlation operation is completed by field programmable gate array (FPGA) where the digital reference cosine signal y(n) is directly generated.

As Figure 4 shows, by detecting the amplitudes $A_{i}^{\left(j\right)}$ of r(m) over $N_{A}$ periods, from Equation (20):

(20) $$A_{beam}^{\left(j\right)}=\frac{2}{cK}\cdot \frac{1}{N_{A}}\sum_{i=1}^{N_{A}}A_{i}^{\left(j\right)}$$

we can estimate the amplitude $A_{beam}^{\left(j\right)}$ corresponding to the energy of the beacon light in the *j*-th (*j* = A, B, C, D) quadrant. Substituting $A_{beam}^{\left(j\right)}$ into Equation (1), the position of the light spot in the QD can be obtained.

## 5. Experiment Results

**Figure 5:**
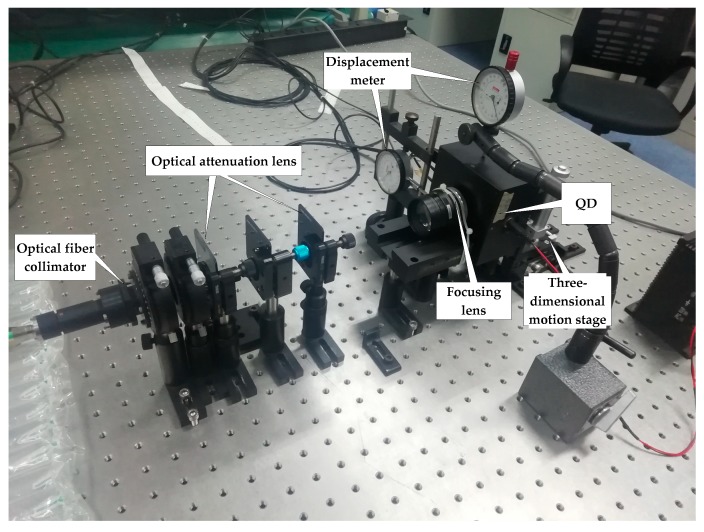
The experimental platform.

The experiment platform based on Figure 4 is shown in Figure 5. The experiment system uses a modulated laser source with a wavelength of 1550 nm, and the output laser is intensity modulated by a cosine signal of 125 KHz. The A/D converter (ADC) output data width is 16 bits, and its sampling frequency is 100 MHz. The QD has an active radius of 1 mm with a gap width of 0.01 mm and is mounted on a three-dimensional micro-displacement motion stage; thus, the relative beacon spot position with respect to the QD can be adjusted. 

When the spot radius was 0.42 mm, 0.53 mm, and 0.71 mm, we respectively recorded the calculated spot positions in two kinds of QD output SNR conditions. The sampling data involved in cyclic cross-correlation are 8000 points. 

For comparison, we also used the direct detection method to detect spot position in the same conditions of spot radius and the QD output SNR. The direct method adopts non-modulation laser light. Then we sampled 800 points of the photocurrent amplitude output from the QD and calculated the mean amplitude value. We finally substituted the mean value in Equation (1) to calculate the spot position.

**Figure 6:**
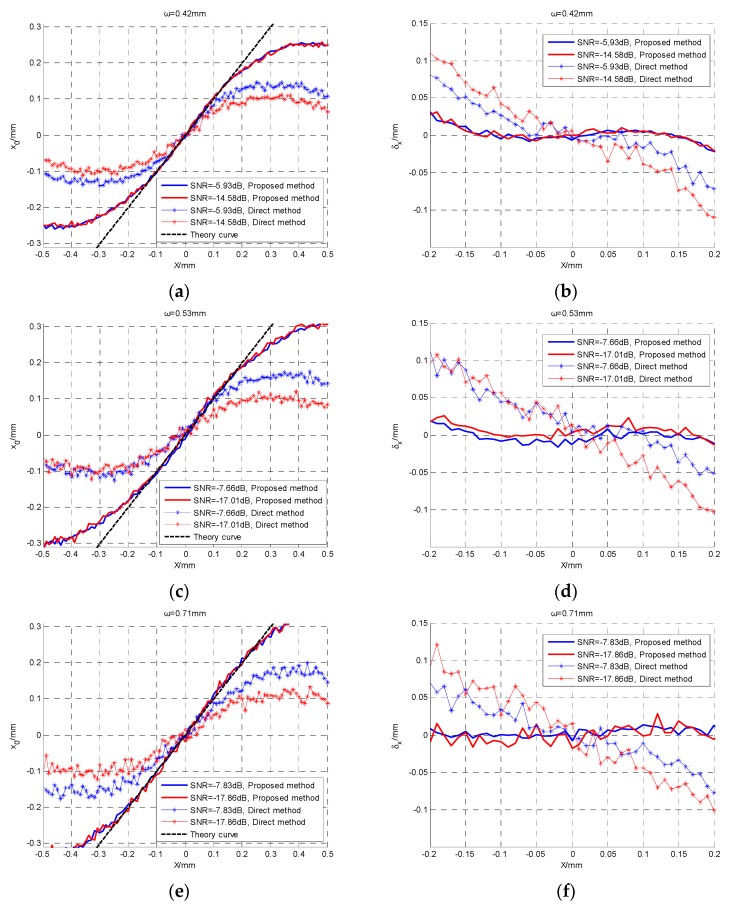
The calculated x coordinate curves and absolute errors of the two methods. (**a**) The calculated x coordinate curves when ω = 0.42 mm; (**b**) the absolute errors when ω = 0.42 mm; (**c**) the calculated x coordinate curves when ω = 0.53 mm; (**d**) the absolute errors when ω = 0.53 mm; (**e**) the calculated x coordinate curves when ω = 0.71 mm; (**f**) the absolute errors when ω = 0.71 mm.

In order to directly compare the performance of the two methods, the absolute errors are calculated. Taking x coordinates as an example, the absolute error is denoted as:

(21) $$\delta_{x}=x_{0}-X$$

where $x_{0}$ is the calculated x coordinate, *X* is the real x coordinate. The experimental results are shown in Figure 6. 

In Figure 6a,c,e, ω is the spot radius, the black dotted line represents the calculated x coordinate curve in the ideal case, the red and blue solid lines represent the calculated x coordinate curves of the proposed method in the two QD output SNR conditions, and the red and blue star lines represent the calculated x coordinate curves of the direct method respectively corresponding to the two output SNR values. In Figure 6b,d,f, the red and blue solid lines represent the absolute error curves of the proposed method in two QD output SNR conditions, and the red and blue star lines represent the absolute error curves of the direct method respectively corresponding to the two output SNR values. 

As Figure 6 shows, with the decrease of the QD output SNR, the calculated x coordinate curves of the proposed method maintain good linearity within the interval (−0.2, 0.2), and the absolute errors are limited within the range of 0.02 mm. In contrast, the linearity of the direct method deteriorates quickly, and its absolute error value increases sharply. 

Furthermore, we use maximum error and root-mean-square error to compare the performance of the two methods. Maximum error is defined as:

(22) $$\delta_{\max}=\underset{i}{\max}(|\delta_{i}|)$$

and root-mean-square error is defined as:

(23) $$\delta_{RMSE}=\sqrt{\sum_{i=1}^{N}\delta_{i}^{2}/N}$$

The performance of the QD is influenced by its own target surface size and gap size, the detected spot position curve presents obvious nonlinearity [1]. To avoid the impact of non-linearity of the QD, the calculated x coordinates in interval (−0.1, 0.1) with good linearity were selected to calculate the two kinds of errors. The comparison of the two methods is shown in Table 2.

It can be seen from Table 2 that the cyclic cross-correlation method is superior to the direct method. In particular, even when the QD output SNR = −17.86 dB, the root-mean-square error of the proposed method is 0.0084 mm and is a quarter of that of the direct method. Moreover, the maximum error is also a quarter of that of the direct method.

Furthermore, the proposed method is insensitive to the change of spot radius. When the QD output SNR is almost −17 dB, with the increasing of spot radius from 0.53 mm to 0.71 mm, the root-mean-square error increases slightly from 0.0069 mm to 0.0092 mm. 

## 6. Conclusions

In conclusion, we discussed the problem that the spot position detection accuracy of the QD decreases significantly in low SNR condition. According to the QD’s working characteristics, we propose a method to improve the spot detection accuracy by cyclic cross-correlation which can enhance the QD output SNR at a frequency point. This method can greatly improve the accuracy of QD position detection in the condition of extremely low SNR. The experiment shows that even when the QD output SNR = −17.86 dB, the root-mean-square error of the proposed method is 0.0092 mm and is a quarter of that of the direct method, and the max error is also a quarter of that of the direct method. Moreover, When the QD output SNR is less than −17 dB, with the increasing of spot radius from 0.53 mm to 0.71 mm, the root-mean-square error increases only 0.0023 mm, which means the proposed method is insensitive to the change of spot size and has strong applicability.

Furthermore, this method only needs a small amount of data to accomplish the calculation, which greatly improves the calculation efficiency and costs little hardware resource. Therefore, this method is especially suitable for real-time spot position detection.

## Figures and Tables

**Table 1:** The changes of signal-to-noise ratio (SNR) with different data lengths.

QD Output SNR (dB)	SNR after Cyclic Cross-Correlation (dB)		
	T = 5	T = 10	T = 20
−7.66	12.46	15.04	15.37
−17.01	12.03	14.19	14.30

**Table 2:** The errors in different spot radius and SNR conditions.

Spot Radius (mm)	SNR (dB)	Proposed Method		Direct Method	
		$\delta_{x\max}$ (mm)	$\delta_{xRMSE}$ (mm)	$\delta_{x\max}$ (mm)	$\delta_{xRMSE}$ (mm)
0.42	−5.93	0.0083	0.0044	0.0272	0.0122
	−14.58	0.0104	0.0048	0.0420	0.0236
0.53	−7.66	0.0101	0.0056	0.0454	0.0150
	−17.01	0.0133	0.0069	0.0565	0.0311
0.71	−7.83	0.0138	0.0067	0.0522	0.0191
	−17.86	0.0183	0.0092	0.0656	0.0352
